# Carbonic anhydrase IX as a novel candidate in liquid biopsy

**DOI:** 10.1080/14756366.2019.1697251

**Published:** 2019-12-02

**Authors:** Ozen Ozensoy Guler, Claudiu. T. Supuran, Clemente Capasso

**Affiliations:** a Department of Medical Biology, Faculty of Medicine, Yildirim Beyazit University, Ankara, Turkey; b Department of NEUROFARBA, Section of Pharmaceutical and Nutraceutical Sciences, University of Florence, Polo Scientifico, Firenze, Italy; c Department of Biology, Agriculture and Food Sciences, Institute of Biosciences and Bioresources, CNR, Napoli, Italy

**Keywords:** Liquid biopsy, cancer, carbonic anhydrase IX, tumour markers, acidification

## Abstract

Among the diagnostic techniques for the identification of tumour biomarkers, the liquid biopsy is considered one that offers future research on precision diagnosis and treatment of tumours in a non-invasive manner. The approach consists of isolating tumor-derived components, such as circulating tumour cells (CTC), tumour cell-free DNA (ctDNA), and extracellular vesicles (EVs), from the patient peripheral blood fluids. These elements constitute a source of genomic and proteomic information for cancer treatment. Within the tumour-derived components of the body fluids, the enzyme indicated with the acronym CA IX and belonging to the superfamily of carbonic anhydrases (CA, EC 4.2.1.1) is a promising aspirant for checking tumours. CA IX is a transmembrane-CA isoform that is strongly overexpressed in many cancers being not much diffused in healthy tissues except the gastrointestinal tract. Here, it is summarised the role of CA IX as tumour-associated protein and its putative relationship in liquid biopsyfor diagnosing and monitoring cancer progression.

## 1. Introduction

### 1.1. Carbonic anhydrases (CAs)

A crucial physiological reaction for the survival of all living organisms is the pivotal CO_2 _hydration/dehydration of the central metabolism. This reaction is connected with numerous metabolic pathways, such as photosynthesis and carboxylation reactions, and biochemical pathways including pH homeostasis, secretion of electrolytes, transport of CO_2_ and bicarbonate, and so on^1^^,^^2^. Moreover, the interconversion of CO_2_ and HCO_3_^−^ is spontaneously and precisely balanced form the living organisms to maintain the equilibrium between dissolved inorganic carbon dioxide (CO_2_), carbonic acid (H_2_CO_3_), bicarbonate (HCO_3_^−^) and carbonate (CO_3_^2−^)^3–6^. The CO_2_ hydration/dehydration is catalysed by a superfamily of metalloenzymes, known as carbonic anhydrases (CAs, EC 4.2.1.1)^7–11^, which are categorised into eight genetically distinct families (or classes), named with the Greek letters: α, β, γ, δ, ζ, η, ɵ, and ι. The last three classes were recently discovered^12–14^. Moreover, members of each class possess multiple transcript variants and protein isoforms, which are characterised by different biochemical properties and have specific tissue/organ and sub-cellular localizations^11^^,^^15–20^. CAs present in animals belong to α-class^21^^,^^22^, plants and algae have α-, β-, γ-, δ- θ- and ι-classes; fungi encode for α- and β-CAs; protozoa for α-, β- and/or η-CAs; bacteria for α-, β-, γ- and ι-CA classes^11^^,^^20^^,^^23–27^. Besides, a matrix protein called nacrein has been identified in the oyster *Pinctada fucata*. It participates in the formation of the nacreous layer and is characterised by a CA domain present at the N-terminus part of the polypeptide sequence^28^. In mammals, 16 α-CA isoforms have been identified: five of them are cytosolic (CA I, CA II, CA III, CA VII, and CA XIII), five are membrane-bound (CA IV, CA IX, CA XII, CA XIV, and CA XV), two are mitochondrial (CA VA and CA VB), and only one is secreted (CA VI), the last three (CA VIII, CA X, and CA XI) being devoid of catalytic activity and referred to as CA Related Proteins (CARPs)^8^^,^^29^^,^^30^. CA active site includes a zinc ion (Zn^2^^+^), which plays a critical role in the catalytic enzyme function. In addition to the zinc, ζ-and γ-CAs reflect exceptions to this principle since they can use cadmium (ζ), iron (γ), or cobalt (γ)^31^^,^^32^.

### 1.2. Tumour-associated CA IX

**Figure 1.**
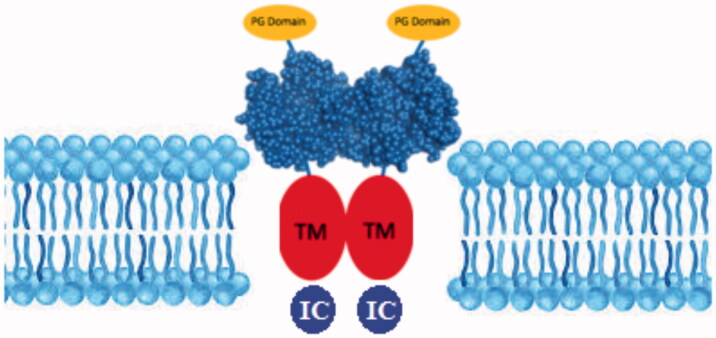
Structure of CA IX isoenzyme. (PG: Proteoglycan domain; TM: Transmembrane domain; IC: Intracellular domain).

**Figure 2.**
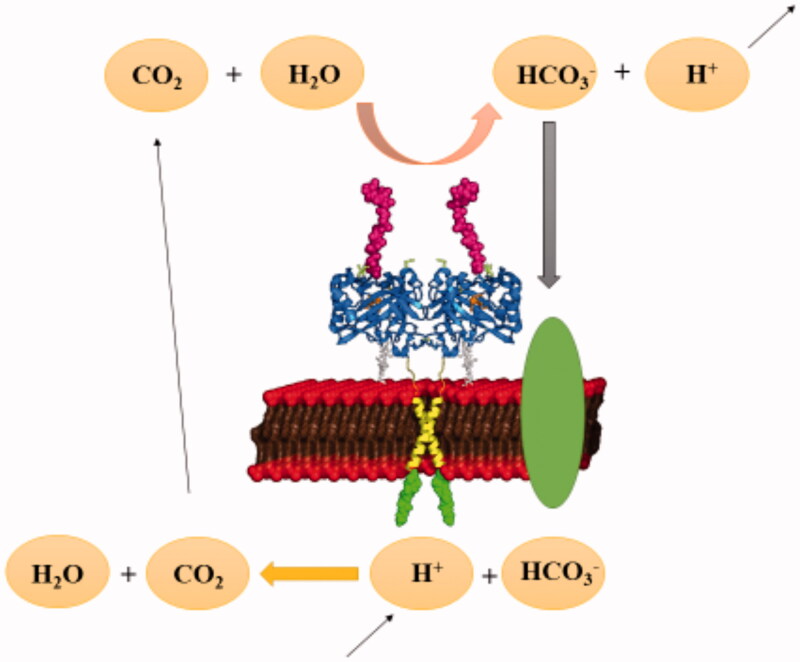
pH regulation of carbonic anhydrase enzymes in tumour cells.

The glycolytic metabolism of cancer was evaluated for so many years to describe the fundamental role of tumour microenvironment and glycolysis in cancer growth and progression^33^. The transcription factors of the glycolytic pathway affect cell proliferation, which is an essential feature of carcinogenesis^34^. Different types of enzymes are produced by tumours or by the body in response to malignancy and used as cancer biomarkers^35^. It has been shown that the expression levels of certain enzymes can vary in various types of cancer^35^. CA IX is a transmembrane CA isoform expressed in healthy tissues. Figure 1 shows the catalytically active CA IX on the cellular membrane surface^36–38^. CA IX is highly overexpressed in many types of cancer^39^^,^^40^. For example, its expression is increased considerably in solid tumours of uterus, kidney, lung, colon, breast, brain, and ovary^41–44^. Tumour cells decrease their extracellular pH by lactic acid production and CO_2_ hydration, which is catalysed by CAs (Figure 2). Since the tumour-associated CA IX is an efficient catalyst for the conversion of CO_2_ in bicarbonate and protons, they contribute to the acidification of the tumour environment. Moreover, its activity leads to the acquisition of metastatic phenotypes and chemoresistance to weakly basic anticancer drugs^21^.

Interestingly, CA IX has a metastatic activity related to the extracellular acidity since it has been shown to promote migration and invasion in tumour cells^45^^,^^46^. Furthermore, oxygen has a crucial function in the regulation of redox balance and energy production in tumour tissues. An inadequate amount of oxygen in the tissues causes hypoxia, which is a characteristic marker of the tumour microenvironment. Hypoxia regulates the expression of many genes inducing a phenotypic alteration of stromal cells in the tumour microenvironment and promoting the survival of cancer cells. Low oxygen activates the HIF-1, the hypoxia-inducible factor 1 (HIF-1)^47^^,^^48^, which starts the transcription of several hypoxia-inducible genes, such as (Vascular Endothelial Growth Factor (VEGF), Glucose Transporter 1 (GLUT1), CA IX and CA XII))^49^. CA IX is one of the most potent hypoxia-induced proteins, and the hypoxia-inducible proteins are important anti-cancer targets^50^. As a result, it is readily apparent that CA IX is associated with many tumours and are involved in the cancer process^34^. The specific inhibition of CA IX activity with selective inhibitors, such as sulphonamide derivatives, represents a good strategy for establishing the driving role of this isoform, as well as other CAs, for example, CA XII, in tumorigenesis^51^. Many CA inhibitors exist, which could be classified as inhibitors binding the metal ion, inhibitors anchoring to the water molecule/hydroxide ion coordinated to the metal, inhibitors occluding the active site entrance and inhibitors linking out of the active site^52^. Many of these inhibitors can be used to reduce the proliferation and invasion capacity of cancer cells^53–55^. Since pH-related cancer growth and metastasis might be dependent on the enzyme activity of the CA IX isoform, the identification of CA IX as tumour biomarker in the liquid biopsy is crucial for a new approach concerning the treatment of malignancy. The role of cancer markers in carcinogenesis is pivotal for early diagnosis, promotion of suitable procedures, and leading right management strategies^56^.

## 2. Tumour-derived components in liquid biopsy

Recently, liquid biopsy has begun an exciting approach in terms of early detection of tumor^57^^,^^58^. The technique, starting from the blood or body fluids, isolates tumour-derived components, such as circulating tumour cells (CTCs), circulating tumour DNA (ctDNA), and extracellular vesicles (EVs), as a source of genomic and proteomic information in patients with cancer^57^. Liquid biopsy is an appropriate technique for correct treatments and figuring out the genetic changes in the tumour. It can be applied to many types of cancer, analysing the blood or body fluids of the patient taken in a non-invasive manner compared to the biopsy and without even detecting the symptoms^59^.

### 2.1. Circulating tumour cells (CTCs)

Thomas Ashworth investigated CTCs in the 1860s, considering that tumour cells could have crossed the vessel wall and enter the bloodstream^60^. These cells are released from primary or metastatic cells. A few steps are required for the metastatic process. First, CTCs are separated from the tumour and incorporated into the bloodstream. The circulating CTCs are then protected from the immune cells to perform extravasation. After that, these cells adapt to the microenvironment of the new tissue and to form metastatic lesions. CTCs are found in circulation as single CTCs or CTC clusters. In the literature, it has been reported that tumour progression and patient survival is correlated with the numbers of CTCs^61^. The cont of CTCs is also useful in the treatment response process. The technics known as immunophenotypic identification of cytokeratin, enzymatic methods, and RT-qPCR (reverse transcriptase quantitative polymerase chain reaction) are used to detect CTCs^62–66^. The cell surface glycoprotein, EPCAM (Epithelial Cell Adhesion Molecule), which is highly expressed in epithelial cancer cells, serves as the primary antigen for CTCs detection. Moreover, with this method, prognostic information can be obtained in metastatic breast, colon, and prostate cancers. CTCsare subjected to genomic mapping^62–66^, allowing a detailed analysis of the genes responsible for the uncontrolled growth of cells. Of course, the major drawbacks of this technique are (i) to distinguish cancer cells from millions of healthy cells; (ii) the detection of the kind of cancer.

### 2.2. Circulating tumour DNA (ctDNA)

The presence of ctDNA in the blood is dated back in 1948s^67^. Although circulating cell-tumour DNA was first identified in 1948, it has only recently been investigated in “liquid biopsy” as cancer biomarkers^68^. Tumours release fragments of DNA into the circulatory system, which are detectable and specific to cancer. There are several advantages to assessing ctDNA. Sampling is non-invasive and inexpensive compared to the tissue biopsy. Besides, ctDNA testing can be easily and frequently repeated to monitor changes that occur during treatment, serving as an early indicator of recurrence, resistance, or metastasis^69^. Liquid biopsy is believed to exhibit tumour heterogeneity, as cells circulate from different regions of the tumour, and to obtain information in a shorter time^69^. CTCs and ctDNAs derived from primary or metastatic cells are abundant in blood. Both CTC and ctDNA provide prognostic information based on the number and level of events detected^70–72^ (Table 1).

**Table 1.** Comparison of CTCs and ctDNA.

Comparision	CTCs	ctDNA
Origin	İntact cells	Released from apoptotic and necrotic cells
Description	Originates from primary tumours or metastasis	DNA fragments in blood circulation
Detection techniques	Density/size based immunomagnetic and microfluidic techniques	PCR or sequencing based
Advantages	Allows for DNA, RNA, protein research Research at single-cell level Captured cells can be used for *in vitro* or *in vivo* studies Clinically validated technology available (CellSearch System)	Easily isolated with kits Can be stored for a long time Gives more precise results Clinically validated for EGFR mutations in non-small cell lung cancer
Disadvantages	A small number of cells are obtained in non-metastatic conditions Can not be stored for a long time Detection steps are expensive	Prognostics and predictability are unclear Only DNA sequence analysis can be performed Known target mutations are needed

### 2.3. Extracellular vesicles (EVs)

Extracellular Vesicles (EVs) are lipid structures released from cells. EVs contain proteins and nucleic acids and play a role in cellular communication, immune regulation, and microenvironmental modulation^73^^,^^74^. Nanosized exosomes (70–150 nm) are the most prominent members of these so-called extracellular vesicles (EVs) and are released from body fluids such as urine, ascites, and plasma^73^^,^^74^. Moreover, they are liberated over all kinds of body cells (epithelium cells, haematopoietic cells, adipocytes, healthy and malignant cells)^75^, released in almost all cell types under physiological and pathophysiological conditions and mediate intercellular contacts^76^. A theranostic solution could be represented by the nanosized EVs, which may transmit biomarkers of diseases and/or vectors of therapeutic molecules, offering a unique opportunity to use a combination of different markers specifically expressed for tumour-derived EVs^74^^,^^76^^,^^77^. For example, Prostate-Specific Antigen (PSA) does not differentiate between benign prostatic hyperplasia (BPH) and a Prostate Cancer (PC), resulting in large numbers of unnecessary biopsies and missed diagnosis of cancer. Since exosomes are directly detectable in patient plasma, the plasmatic exosomes expressing PSA have the potential in distinguishing healthy individuals, BPH, and PC^77^. Recently, it has been demonstrated that neurodegenerative disorders, including Alzheimer’s disease (AD), Parkinson, and amyotrophic lateral sclerosis, are correlated with extracellular vesicles. EVs have also been investigated in relation to infection caused by viruses, bacteria, fungi, protozoa, and helminths^76^. Such pathogens secrete EVs, and prions were even present in EVs.Finally, EVs seem to play key roles in autoimmune diseases^76^. EVs circulating in body fluids are valuable liquid biopsy biomarkers. Additionally, their protein concentration is higher in patients with advanced tumours^73^.

## 3. Tumour-associated CA IX as biomarker in liquid biopsy

The tumour-associated CA IX may be used as a cancer biomarker in the liquid biopsy technique. Carbonic anhydrase IX is a transmembrane enzyme^78^, and it is involved in the growth and development of tumour cell adhesion^79^^,^^80^. There is an association between elevated serum levels of CA IX and CTCs^47^. This suggests a relationship between hypoxia and tumour cell circulation in the bloodstream. In the peripheral venous blood, it is also possible to find the soluble form of CA IX, which is released by proteolytic cleavage^47^^,^^50^^,^^81^. For example, a high level of soluble CA IX was found in the serum of patients with renal cancer^50^. Müller et al. investigated the relationship between serum levels of CA IX and CTCs in metastatic breast cancer^47^. Their findings suggested that the CA IX activity level was higher in cancer types with a high number of CTCs. In this condition, it is expected a decrease in the patient’s overall survival. Besides, CA IX disrupts cell-cell and cell-matrix interactions by triggering tumour acidification. As a consequence, CTCs separate from the primary tumour, and invasion occurs. Probably, CA IX inhibition could slow down the invasion process of CTCs.

During the tumour progression, exosomes and the metalloenzyme CA IX affect the growth and proliferation of the tumour. The relationship between exosomes and CA IX has been investigated using an *in vitro* cellular model of human prostate carcinoma cell line cultured in different pH conditions. The results showed that the acidic microenvironment increased both the expression and activity of CA IX in cancer cells^82^. Besides, the number of exosomes released by the cancer cells was raised together with the upregulation of the CA IX^82^. These data strongly support that exosomes and CA IX are tumour-associated components and the enzyme CA IX is a cancer biomarker that could be used as valuable target of the liquid biopsy^82^. Horie et al. demonstrated that CA IX exosomes were released from renal carcinoma cells^49^. The quantity of exosomal CA IX is increased in hypoxia response, promoting upregulation of MMP-2, migration and tube formation, and may induce angiogenesis in the tumour microenvironment^49^. Dorai and co-workers analysed the effect of increasing expression levels of CA IX in renal cancer cells. They showed that the level of released gangliocytes was positively correlated to that of exosomal CA IX expression^83^. Gangliosides play a critical role in cell adhesion, migration, and cell signalling^84^. Since CA IX induces the release of gangliocyte-containing exosomes, the exosomal CA IX may be a valuable biomarker of the carcinogenesis process.

CA IX expression is regulated exclusively by HIF-1α, rapidly increases in response to hypoxia, and is very important for maintaining the acidic pH of the tumor^85^. Brown-Glaberman proposed that the circulating CA IX could be considered as a biomarker for detecting the level of hypoxia and the upregulation of HIF-1α^86^^,^^87^. Circulating CA IX can be easily isolated from body fluids and considered a biomarker for different stages of cancers and the differentiation of local/advanced tumours. Malentacchi and coworkers to validate circulating CA IX as a tumour biomarker measuring the CA IX mRNA in the urine sediments of patients affected by kidney, prostate, and bladder cancers^88^. As a result, they associated the mRNACA IX expression in the tumour of urogenital origin. Liu et al. have found that the combination of the CA IX/CD147 antibodies achieved higher efficiency in the NanoVelcro platform compared to EPCAM-based methods for capturing circulating cells coming from the renal carcinoma^89^.

## 4. Conclusion

Liquid biopsy technology allows the detection of solid tumours, such as those involving lung, breast, and pancreatic, using the blood or other body fluids. Liquid biopsy can detect cancer-specific markers even in lesions that are too small to be recognised by other available methods, indicating that this method can be used early in cancer diagnosis. Among the known biomarkers of the liquid biopsy, the CA IXisoenzyme could be a promising candidate for tumour detection. In fact, the CA IX activity level is higher in cancer types with a high number of CTCs; the quantity of exosomal CA IX is increased in hypoxia response; the release of the exosomal gangliocyte is correlated to the exosomal CA IX expression increase; the circulating CA IX is associated to tumors of urogenital origin; CA IX disrupts cell-cell and cell-matrix interactions by triggering tumour acidification. In this context, the tumour-associated CA IX could be considered a valid biomarker of the non-invasive liquid biopsy, which is viewed as a technique that offers future research on precision diagnosis and treatment of tumours in a non-invasive manner. Moreover, CA IX could be a valid molecular target in the treatment of cancer.
